# Development of Soft Wrinkled Micropatterns on the Surface of 3D-Printed Hydrogel-Based Scaffolds via High-Resolution Digital Light Processing

**DOI:** 10.3390/gels10120761

**Published:** 2024-11-23

**Authors:** Mauricio A. Sarabia-Vallejos, Scarleth Romero De la Fuente, Nicolás A. Cohn-Inostroza, Claudio A. Terraza, Juan Rodríguez-Hernández, Carmen M. González-Henríquez

**Affiliations:** 1 Facultad de Ingeniería, Arquitectura y Diseño, Universidad San Sebastián, Santiago 8420524, Chile; mauricio.sarabia@uss.cl; 2 Departamento de Química, Facultad de Ciencias Naturales, Matemáticas y del Medio Ambiente, Universidad Tecnológica Metropolitana, Santiago 7800003, Chile; scarleth.romerod@utem.cl; 3 Instituto Universitario de Investigación y Desarrollo Tecnológico (IDT), Ignacio Valdivieso 2409, Santiago 8940000, Chile; nicolas.cohn@utem.cl; 4 Research Laboratory for Organic Polymer (RLOP), Facultad de Química y Farmacia, Pontificia Universidad Católica de Chile, Santiago 7810000, Chile; cterraza@uc.cl; 5 Polymer Functionalization Group, Departamento de Química Macromolecular Aplicada, Instituto de Ciencia y Tecnología de Polímeros-Consejo Superior de Investigaciones Científicas (ICTP-CSIC), 28006 Madrid, Spain; jrodriguez@ictp.csic.es

**Keywords:** 3D porous scaffolds, stereolithography, wrinkled nonplanar surfaces

## Abstract

The preparation of sophisticated hierarchically structured and cytocompatible hydrogel scaffolds is presented. For this purpose, a photosensitive resin was developed, printability was evaluated, and the optimal conditions for 3D printing were investigated. The design and fabrication by additive manufacturing of tailor-made porous scaffolds were combined with the formation of surface wrinkled micropatterns. This enabled the combination of micrometer-sized channels (100–200 microns) with microstructured wrinkled surfaces (1–3 μm wavelength). The internal pore structure was found to play a critical role in the mechanical properties. More precisely, the TPMS structure with a zero local curvature appears to be an excellent candidate for maintaining its mechanical resistance to compression stress, thus retaining its structural integrity upon large uniaxial deformations up to 70%. Finally, the washing conditions selected enabled us to produce noncytotoxic materials, as evidenced by experiments using AlamarBlue to follow the metabolic activity of the cells.

## 1. Introduction

There is an urgent need to find different ways to replace organs or tissues damaged due to a particular disease, trauma, or congenital problems. Cutting-edge medical treatments and therapeutic techniques address these challenges by enabling the development of tissue engineering procedures. Modern medicine uses organ transplantation to repair damaged tissues, especially allografts and autografts [1]. However, these transplants have important disadvantages like infectious disease transmission and the underlying danger of inducing an adverse immunological response in the patient. By employing artificial scaffolds, tissue engineering has developed as an alternative methodology to repair and regenerate injured tissues that surpasses the drawbacks of existing supplies and techniques. Scaffolds are designed to assist in the reconstruction and regeneration processes of damaged tissues by serving as templates that ensure the optimal conditions for cell proliferation, differentiation, and survival [2].

The replication of the extracellular matrix, or ECM, is a central concept underpinning scaffold architecture. This guarantees that the scaffolds’ design and mechano-biological activities closely mimic those of natural tissues [3]. Scaffolds must have the necessary porosity to allow vascularization, new tissue formation, remodeling, and integration with host tissue after implantation. They should offer stability and adaptability regarding mechanical strength and shape to facilitate the repair of the defective tissue. The main problem with including porosity in the scaffold is that removing material from the piece reduces its mechanical performance. This trade-off between porosity and mechanical resistance must be perfectly balanced and optimized to obtain an ideal scaffold [4,5].

Scaffold hierarchy is a crucial requirement in this scenario. To fulfill their innate structure–function relationship, natural tissues must be organized at multiple levels, starting at the nano- or micro-scale and scaling to the macro-organization of the tissue. For example, at large-scale organization (macrostructural), the bone includes two types of tissues: compact (cortical) and spongy (trabecular). Cortical bone provides strength, while trabecular bone contributes to lightweight support and permits tissue perfusion due to its large porosity [6]. At the microstructural level, the osteons in cortical bone and the trabeculae in spongy bone form networks that contribute to the bone’s resilience [7]. At the nanoscopic level, bone tissue presents mineralized collagen fibrils—hydroxyapatite nanocrystals embedded within collagen. This hierarchical structure permits the bone tissue to combine the rigidity and flexibility necessary for the large cyclic loads that occur physiologically [6]. Also, this highly hierarchical arrangement allows bone to achieve strength, durability, and the ability to remodel in response to mechanical stress or injury.

As a result, bone scaffolds need to have characteristics as similar as possible to those exhibited in natural tissues. These include a macro-scale architecture providing overall structural integrity and mechanical resistance, micro-scale porosity facilitating cell migration and nutrient flow, and nano-scale features replicating the mineralized collagen fibrils. The multi-scale design enhances the scaffold’s ability to support osteogenesis, angiogenesis, and eventual tissue integration [8].

Numerous ways exist to create these multi-scale designs for tissue engineering applications [9,10,11]. In this context, a novel methodology in recent years has involved developing a highly controlled and repeatable porous architecture based on triply periodic minimal surface (TPMS) structures. Despite their complexity, these structures are easily fabricated and can mimic the multi-scale organization of natural bones, from the nano-scale mineralized collagen fibrils to the macro-scale overall framework, enhancing the scaffold’s mechanical strength and biological performance [12]. TPMSs has currently extensively been investigated and has been preferred over conventional scaffolds due to their smooth surfaces (zero local curvature), highly interconnected structures, and the ability to control porosity precisely. These crucial parameters promote cell proliferation, nutrient flow, and vascularization [13]. These biomimetic structures can be found in natural architectures such as leaf surfaces and butterfly wings [14].

One of the most used technologies to fabricate complex scaffolds for tissue engineering nowadays is additive manufacturing (AM), commonly known as 3D printing. AM can be categorized into seven types according to ASTM [15]. Digital light processing (DLP) is particularly interesting due to its precise control over scaffold design and high resolution, enabling the creation of intricate structures with complex internal and external architectures that mimic natural tissues, such as TPMS. DLP and adapted photosensitive hydrogel-based resin are required to fabricate this structure. In this work, scaffolds were fabricated using a biocompatible hydrogel-based resin specifically designed for DLP printing, utilizing reversible addition-fragmentation chain transfer (RAFT) polymerization. This polymerization technique was chosen because DLP printing typically employs conventional radical photopolymerization methods compatible with various monomers and functional groups. However, traditional radical polymerization results in inert or “dead” polymers that cannot be further modified [16]. Instead, RAFT polymerization incorporates agents that allow the reversible deactivation of propagating radical species, making it more adaptable and allowing the incorporation of new monomers or terminal functional groups into an existing polymer network [17].

One of the main issues with RAFT synthesis is its intolerance to environmental oxygen, making the polymerization process challenging in open environmental conditions. Consequently, an efficient photoRAFT system that can withstand oxygen provides an interesting advantage in developing a system that can freely function in an open-air setting to effectively photopolymerize resins [18,19]. Recently, various methods have been used to achieve oxygen-tolerant controlled radical polymerization synthesis mechanisms, including the photoelectron/energy transfer RAFT polymerization (PET-RAFT). In PET-RAFT, it is necessary to use a photoredox catalyst with a high photo-reducing capability, which can convert triplet oxygen into singlet oxygen states, thus allowing the trapping of oxygen during polymerization, avoiding the oxidation of radical groups during the propagation stage [20]. In addition to the 3D structure of the scaffold, another important aspect needs to be added, i.e., controlling both the surface functionality and microstructure of the scaffold. As described in this manuscript, this can be achieved by incorporating wrinkled surfaces on TPMS scaffolds. These wrinkled patterns may significantly improve cell attachment, proliferation, and differentiation by providing both chemical and topographical cues like those found in the ECM [21]. These wrinkles can be formed through various methods, such as mechanical stretching, surface tension modulation, or differential swelling of bilayer or gradient materials [22]. According on the present work, wrinkles derive from surface instabilities in the material surface, which can be induced through either chemical treatments or thermal processes. Differential swelling, on the other hand, occurs when two layers of material with different swelling capabilities (or a single material with gradient properties in this particular case) expand to a different extent upon exposure to a solvent, leading to wrinkle formation due to the tensile mismatch created at the interface. This process can produce various nano- and micro-scale surface morphologies with specific patterns and distributions. More interestingly, this method allows the creation of intricate patterns, such as wrinkles, folds, creases, and ripples, which are challenging or impossible to achieve with traditional micro-modification techniques [23].

One of the main challenges with this wrinkle formation is the possibility of generating these micropatterns over nonplanar structures. To the best of our knowledge, only a few examples have been reported in the recent literature [23]. These microstructured nonplanar surfaces are highly desired due to their enhanced response to particular external stimuli, creating more flexible devices suitable for soft electronic and haptic engineering. There are several possible applications where these nonplanar surfaces can be used like smart optics [24], wearable tactile devices, micropatterns to control friction [25], refreshable Braille surfaces [26], adjustable wettability [27], smart adhesion [28], and stretchable devices [29], among others. In the biomedical area, these nonplanar wrinkled patterns were introduced to improve the antibiofouling properties of the coating [30] or to enhance cell attachment and proliferation over the printed scaffolds [22,31], to mention a few applications.

In this paper, we describe the synthesis of DLP resins based on the common components of hydrogel composites, like dimethylamino ethyl methacrylate (DMAEMA), acrylamide (AAm), and polyethylene glycol diacrylate (PEGDA). DLP technology was used to fabricate the 3D parts through photopolymerization based on PET-RAFT as a synthesis route. The design of the scaffolds focused on the TPMS structures to create an interconnected porous network that, as mentioned above, served to mimic, at least to some extent, the ECM structure, thus facilitating both vascularization and nutrient flow. By taking advantage of the residual liquid film formed on the surface of the printed part, wrinkled patterns were generated using a vacuum, light-induced vitrification, and Ar-plasma exposure. These patterns formed spontaneously due to the strain mismatch present in the material. Once the TPMS structures were printed and wrinkled patterns formed on the top, the obtained 3D parts were used to test the mechanical and cytocompatible performance of the material.

## 2. Results and Discussion

### 2.1. Strategy Proposed for Fabrication of Scaffolds: Design of Photopolymerizable Resin for PET-RAFT Photopolymerization

**Figure 1:**
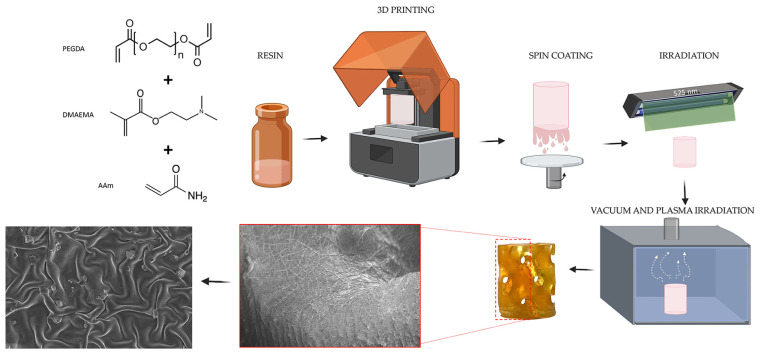
Schematical description of the process followed to obtain the wrinkled scaffolds.

The strategy for preparing porous scaffolds with controlled chemical composition and topographical features is depicted in Figure 1. Briefly, the selected resin compositions were employed for the fabrication of the scaffolds by using high-resolution DLP. Then, upon spin coating, a thin layer (of unpolymerized resin) was obtained at the surface of the scaffold (also in those intricate areas). Finally, depending on the treatment applied, either planar surfaces (directly after curing) or wrinkled surfaces (following vacuum and plasma treatment).

As will be discussed later, this study focused on a photosensitive hydrogel-based resin that is polymerized via a PET-RAFT synthesis route using green irradiation (λ = 525 nm) to create wrinkles on the surface of a 3D material. Surface wrinkling allows the creation of modified topographies with enhanced cellular osteogenesis applications, particularly for bone graft implants.

The subsequent sections of this manuscript are organized as follows: Firstly, two different analyses were performed on the photosensitive resins to analyze their printability, i.e., contact angle (surface tension) and rheological tests (to calculate the Bond number). Secondly, two types of pieces were printed: flat circular pieces as prototypes for printing parameter optimization and for carrying out cytocompatibility tests and three-dimensional scaffolds with internal porous architectures using the TPMS approach for mechanical tests and topographical characterization. Both structures were superficially modified to generate wrinkled patterns on the top of the material. Physicochemical characterization was carried out before and after polymerization (liquid and polymerized resin) to identify the chemical structural changes due to the polymerization process. Additionally, surface analyses like optical microscopy, field-emission scanning electron microscopy (FE-SEM), and atomic force microscopy (AFM) were performed to ensure the homogeneous distribution of wrinkles on the surface of the materials. Finally, mechanical and cytocompatible studies were conducted to corroborate their properties and compare them with natural bone tissue.

### 2.2. Evaluation of the Resin Printability and Selection of the 3D-Printing Parameters

The first step was to characterize the photosensitive resins to evaluate their printability (Table 1). From a rheological point of view, although no specific formula or factor indicates clearly whether a resin is suitable for printing via DLP technology, some key aspects must be taken into account like viscosity and flow properties, which are crucial for evaluating its potential performance as “ink” [32,33]. Ideally, in photoresins employed for DLP, the dynamic viscosity must not surpass 5 × 10^3^ mPa·s; lower viscosities allow printing because a new resin layer could be placed over polymerized faster than the previous one [34]. Additionally, there is a dimensionless number known as the Bond number (B_0_), which is used to compare the gravitational forces and the forces related to the surface tension of the fluid. This number determines if the liquid bridge formed in the interface between the metallic plate and the resin surface is stable. A high value of B_0_ indicates that the system is relatively unaffected by surface tension effects.

In contrast, a low value (typically less than one) suggests that the surface tension of the fluid dominates the system, thus forming stable and axisymmetric liquid bridges. In our case, the dynamical viscosity, surface tension, density, and B_0_ values are tabulated in Table 1 for each analyzed resin. They all resulted in low B_0_ values, indicating stable liquid bridge formation. For more information about the Bond number calculation and its implications, please see references [35,36].

A key factor that significantly influences the printing process in DLP technology is the adhesion force between the resin and the printing plate. This adhesion can be indirectly assessed by measuring the contact angle between the resin and the printing plate. Generally, a low contact angle indicates high surface energy at the interface, suggesting stronger adhesion. Achieving optimal adhesion is essential for producing high-quality prints and minimizing defects. All contact angles between the metallic plate and the resin were lower than 15°, indicating high adhesion forces.

After completing the rheological characterization and confirming the suitability of the resins for DLP 3D printing, the printing parameters to produce the scaffolds were investigated. For this purpose, chemical analysis of the materials obtained was conducted by FT-IR. This spectroscopic technique allowed us to follow the vibrational bands of the characteristic functional groups in the resin. More precisely, both the liquid resin (unpolymerized) and the 3D-printed piece (polymerized) were characterized in each case. These analyses were performed to understand the polymerization process and the consumption of reactive double bonds during the polymerization in the short time employed to polymerize each layer.

**Figure 2:**
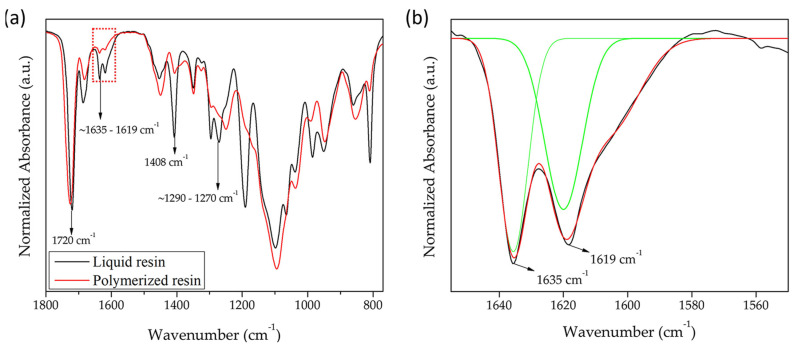
(**a**) FT-IR spectra of liquid and polymerized resin for sample 300:50:150, and (**b**) magnification of vinyl bands (C=C, 1619–1635 cm^−1^, red dashed box) where Gaussian fitting (green line) of the peaks is depicted.

Figure 2a shows the FT-IR spectra of a representative sample (300:50:150) in both states (liquid and polymerized resin). Several characteristic bands are present in the spectra, like the C=O stretching mode (near 1720 cm^−1^) and the C=C stretching mode of the vinyl group (two bands at 1619 cm^−1^ and 1635 cm^−1^). As it was possible to observe, the vinyl band (C=C) decreased in intensity for the liquid resin (unpolymerized) when compared to the printed piece (polymerized) due to the double-bond rupture during polymerization, transforming the double bonds into single bonds. Similarly, the bands located at 1408 cm^−1^ correspond to the CH_2_ scissoring of the CH_2_=CHR mode, while the bands located at ~1270–1290 cm^−1^ correspond to different vibrational bands like CH_2_=CHR of CH rocking, C-O(H) bending, -(CH_2_)_n_- in phase twist, or OCH_2_ wagging mode. Both bands (1408 cm^−1^ and ~1270–1290 cm^−1^) correspond to the vibrations of double bonds. Both showed a significant reduction in intensity when the material polymerized. In the case of the C=O (1720 cm^−1^), the intensity of the peak did not change, as it was expected, because the ester group did not interact with the others during the polymerization process.

By using the intensity of the FT-IR bands, it was possible to monitor the polymerization reaction of the monomeric mixture by tracking the variation in double-bond intensity from the methacrylate and acrylate functional groups (C=C stretching mode, 1619 cm^−1^ and 1635 cm^−1^). Thus, the conversion of vinyl bonds was determined from the normalized area under these vibrational bands using the following equation:

(1) $$Cv=(1-\frac{{{(I}_{C=C})}_{p}}{{\left(I_{C=C}\right)}_{l}})$$

where ${{(I}_{C=C})}_{p}$ and ${\left(I_{C=C}\right)}_{l}$ represent the area under the vibrational band of the C=C group after polymerization (printed part) and the liquid resin (unpolymerized), respectively. In Figure 2b, it is possible to observe a magnification of the zone between 1550 cm^−1^ and 1650 cm^−1^, where the curves fitted (green line) for both peaks are depicted, together with the sum of the peaks (red line) and the raw data (black line). The results demonstrate that all samples presented similar conversion values after 3D printing (C*v*, close to 73–84%). This indicated that between 16% and 27% of the monomers/crosslinking agents remained unreacted; therefore, a postcuring step was required [37]. The subsequent results demonstrated, using the same method, that after a long postcuring step, less than 2% of the monomers/crosslinking agents remained unreacted. According to the literature, polymers containing DMAEMA [38,39,40], PEGDA [41,42], and AAm [43,44] have shown no cytotoxicity after exposure periods ranging from 48 h to 7 days. This study assessed the long-term effects (7 days) on cell proliferation, revealing sustained metabolic activity in cells exposed to the tested materials.

### 2.3. CAD Models Employed for Scaffold Fabrication

**Figure 3:**
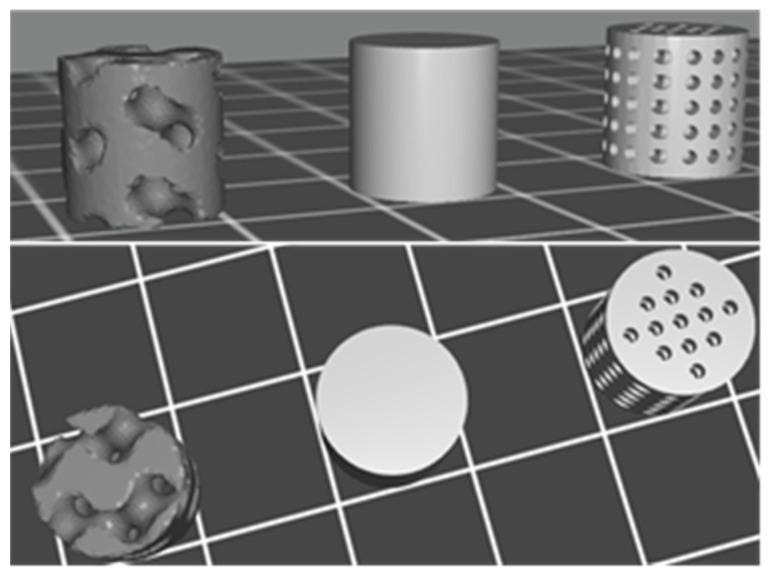
STL representation of the CAD models used for test 3D-printed scaffolds: gyroid-TPMS (**left**), solid (**center**), and straight cylindrical channels (**right**).

Three different internal scaffold structures were tested in this study to generate porous media: gyroid (TPMS), solid scaffolds, and straight cylindrical channels [45]. TPMSs are smooth porous surface models with the distinctive property of infinite repetition in the three spatial directions with a zero mean curvature at every point [46]. This property of TPMSs avoids the generation of the so-called “stress raisers”, which correspond to zones in which shear stresses collide and accumulate, thus producing increasing growth in deformation and stress, leading to fractures and, eventually, the failure of the structure. Figure 3 shows the CAD models used in this study.

Before advancing to the mechanical performance of the material and to ensure the piece’s noncytotoxicity, the printed samples were immersed in a culture meduim containing phenol red, a reagent known to rapidly change color in response to acidity (yellow) or alkalinity (fuchsia). This preliminary test allowed us to discard the compositions (resins) that generated important pH changes in the media. In our case, the resin with a mole ratio of 300:50:150 stood out from the rest as the most suitable for biomedical applications due to its null response to phenol red, not producing any change in the medium color, indicating that there was no delivering of components from the material to the media. Based on these findings, for the cytocompatible and mechanical tests, we exclusively used the 300:50:150 resin for future investigations since this resin was the most appropriate for producing biomedical devices.

### 2.4. Fabrication and Evaluation of the Mechanical Properties of the 3D-Printed Scaffolds

**Figure 4:**
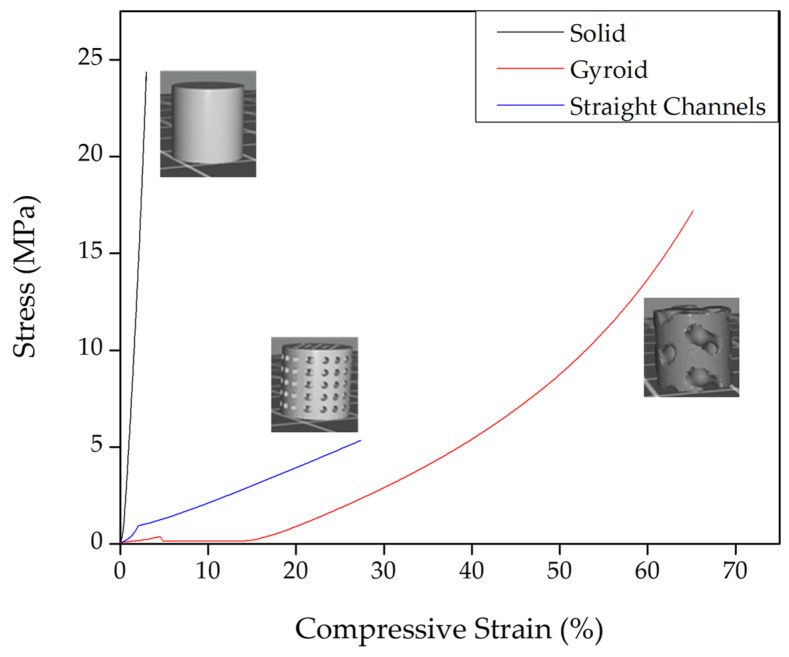
Stress–strain curves from the compressive mechanical tests of the three different structures studied.

As mentioned, the compression velocity was kept sufficiently slow to measure the material under a quasistatic regime (strain rates below 10^−1^ s^−1^). This approach ensured that the mechanical parameters provided a nondynamic representation of the material. Maintaining a low compression velocity can minimize the influence of rate-dependent factors, providing a clearer understanding of the material’s intrinsic mechanical properties. This method is particularly valuable for applications where the material will be subjected to static or slowly varying loads, allowing for more precise and relevant mechanical characterization. The results for sample 300:50:150 are shown in Figure 4; these values correspond to the mean of five samples.

The black line in Figure 4, which corresponds to the solid model, is the one that presents the highest mechanical resistance, 9.08 ± 0.56 MPa, according to Table 2. This was an expected result because it was the model with a higher amount of material (0% porosity), in contrast to the straight channels and the gyroid models, which both possessed 40% porosity (60% material, 40% void, according to the CAD model). In the case of the gyroid model, it is possible to observe that the mechanical resistance was slightly higher than that of the straight channel model (0.51 ± 0.05 MPa versus 0.36 ± 0.07 MPa). Still, the maximum deformation at break was considerably higher, almost tripling the compressive strain values obtained, from 27.7% to 66.8%, from the straight channels to the gyroids, respectively. A similar situation occurred with the maximum stress at fracture, which also tripled in value (from 5.53 ± 2.45 MPa to 17.08 ± 2.97 MPa). This behavior indicated that by altering the internal architecture of the scaffolds, it was possible to manipulate the mechanical performance of the piece by avoiding the stress concentration in the structure, making it possible to bear higher mechanical solicitations more efficiently without compromising the integrity of the scaffold.

### 2.5. Surface Characterization of the 3-Printed Scaffolds

After analyzing the resin’s printability, the optimal parameters for 3D printing, and the mechanical performance, the surface micropatterns generated over the 3D-printed parts were characterized. The procedure used to generate wrinkled micropatterns included five steps: (1) spin coating, (2) short green-light exposure, (3) vacuum exposure, (4) Ar-plasma exposure, and (5) postcuring green-light exposure.

**Figure 5:**
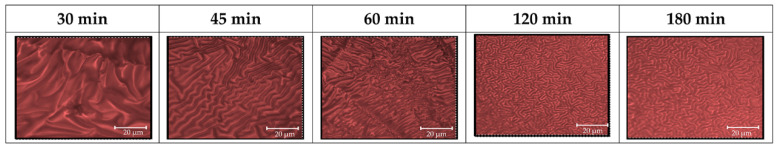
OM images of the surface wrinkled micropatterns obtained at different vacuum exposure times from 30 min to 180 min.

The first parameter we adjusted to generate different patterns was the vacuum exposure time, which varied from 30 to 180 min (Figure 5). The obtained samples were then examined using optical microscopy. As it is possible to observe, longer exposure times produced a reduction in wrinkle dimensions; in particular, the wrinkle wavelength (width of the wrinkle) reduced from 11.6 ± 0.5 µm to 4.1 ± 0.3 µm (from 30 min to 180 min), indicating that, at least to some extent, it was possible to control the surface microstructure of the printed parts simply by altering the process formation parameters like vacuum exposure time. Similar studies varying the green light and Ar plasma exposure were also performed on the samples, concluding that the wrinkle size dimension was not significantly affected by varying these parameters. These results agree with previous results [37] carried out in chemically different resin-based hydrogels (HEMA-PEGDA).

We also analyzed the surface polarity behavior of the printed parts. To do this, both wrinkled and planar (nontreated) discs were printed using DLP technology to test their wettability against different liquid media. To determine the surface free energy (SFE) of the material, contact angle measurements were carried out using solvents with different polarities but with similar surface tensions: water (polar), glycerol (mid-polar), and diiodomethane (apolar). Using these results and Equation (2), it was possible to determine the SFE of the printed parts.

As it is possible to observe from Table 3, the material’s polar component (γ_SP_) was similar for both the smooth and wrinkled samples, thus indicating that, independent of the microstructure, the material polarity did not change. Regarding the wrinkled surface, the SFE tended to increase compared to the smooth surface (from 54.57 dyne/cm to 64.89 dyne/cm), indicating a slight increase in surface reactivity, probably due to the increased surface area exposed to the media due to the folds of the wrinkled surface. This analysis demonstrates that wrinkled pattern generation effectively produces physical changes in how the material behaves with the environment [47,48].

**Figure 6:**
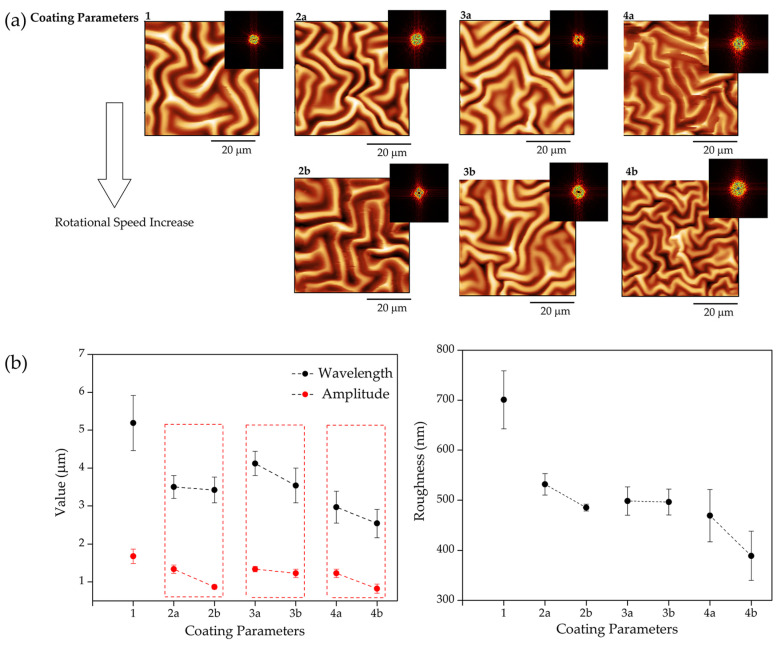
(**a**) AFM micrographs and FFT image; (**b**) wavelength (black line) and amplitude (red line) of the wrinkled patterns and roughness of the samples using different times and speed rotations via spin coating, according to Table 6 for sample 300:50:150.

AFM was conducted to characterize the surface micropatterns and determine the wrinkles’ dimensions, such as height (amplitude) and width (wavelength). To perform these analyses, height profiles were extracted from different sample sectors. These profiles showed a sinusoidal shape, so the wrinkles’ height and width corresponded to the profile’s amplitude and wavelength. As previously discussed regarding the formation of surface wrinkles, a spin-coating method was employed to uniformly distribute the unpolymerized resin across the scaffold’s surface (see Table 6), ensuring consistent and controlled application of the unpolymerized resin for subsequent structural modifications. It was observed that when employing low spin-coating speeds (approximately 1000 RPM), a thin layer of liquid resin (remnant material after 3D printing) was generated on the surface of the printed parts at the edge of the samples. This thin layer was not uniform, thus forming a thicker layer on the center of the printed part, producing “bigger” wrinkles in that region and “smaller” ones close to the edge. This phenomenon highlights the significance of spin-coating parameters in controlling the size and distribution of surface features, offering insights for optimizing the fabrication process. With an increase in rotational speed (approximately 3000 RPM), a layer of liquid resin was uniformly distributed across the entire surface of the part. This homogeneous distribution facilitated the wrinkled pattern formation with consistent dimensions throughout, indicating the critical role of the spin-coating parameters in achieving reproducible surface features. Figure 6 shows the results for the different rotational parameters used for spin coating.

As can be observed in Figure 6a, AFM micrographs were obtained (sample 300:50:150) under different coating parameters (according to Table 6). In general, the application of a second cycle (comparing coating parameter 1 with 2, 3, and 4) heavily affected wrinkle size (Figure 6b), but increasing the rotational speed of the second cycle also affected wrinkle dimensions, forming wrinkled patterns with smaller dimensions (wavelength and amplitude) and roughness. As the spinning speed increased, the layer thickness decreased, thus creating smaller and homogenously distributed wrinkled patterns. Several studies from our research group and external ones have demonstrated that thinner layers produce smaller wrinkles [22,49,50,51,52]. As Rodriguez-Hernandez stated, “the wrinkle wavelength depended linearly with the skin depth” [22], and, in our case, the film thickness and the skin depth depended on the spin-coating parameters used and the surface treatment conditions.

In conclusion, according to previous research group results, the most suitable wrinkle dimensions for generating optimal biocompatible (and antibiofouling) capacity are 1.8–3.0 µm in wavelength and 0.8–1.2 µm in amplitude, thus indicating that the samples fabricated with the following coating parameters, first cycle 10 s at 1500 RPM and second cycle 10 s at 3000 RPM (4a and 4b according to Table 6), were the most suitable for use as a scaffold with cytocompatible characteristics.

**Figure 7:**
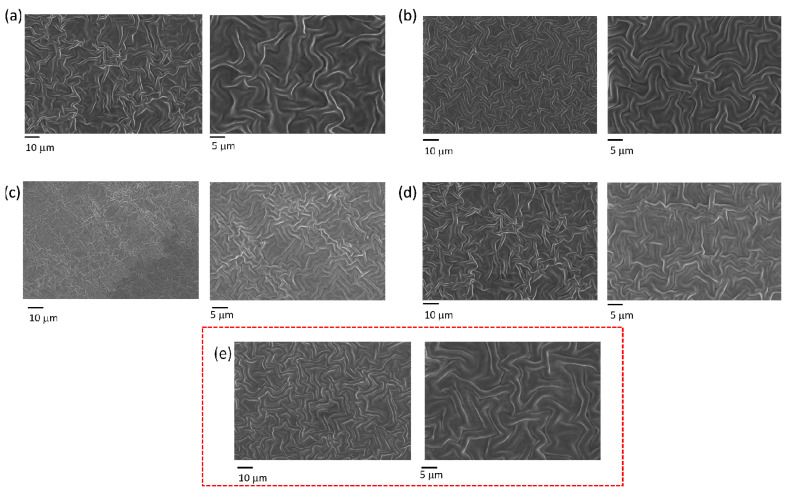
FE-SEM micrographs of wrinkled surfaces with different compositions: (**a**) 500:0:0, (**b**) 400:100:0, (**c**) 400:0:100, (**d**) 300:100:100, (**e**) 300:50:150 of PEGDA:DMAEMA:AAm.

FE-SEM micrographs were also obtained to corroborate the AFM results. The resins were tested with constant spinning parameters (Table 6, 4a and 4b). These results can be observed in Figure 7, and, from its analysis, it is possible to conclude that the resin composition did not significantly affect wrinkle dimensions or distribution (Figure 7a–e). In all cases, the wrinkled surfaces had a labyrinth-like pattern with evenly distributed wrinkles of similar sizes. As mentioned before, the 300:50:150 sample (Figure 7e, with red dashed square) was selected for the final cytocompatibility analyses due to its remarkable performance in the phenol red test used as the preliminary selection method. For the sample 300:50:150, the average wavelength was 2.55 ± 0.28 μm, the average amplitude was 0.86 ± 0.05 μm, and the roughness as 381.72 ± 10.41 nm.

### 2.6. Cytocompatibility Evaluation of the Designed Resins

**Figure 8:**
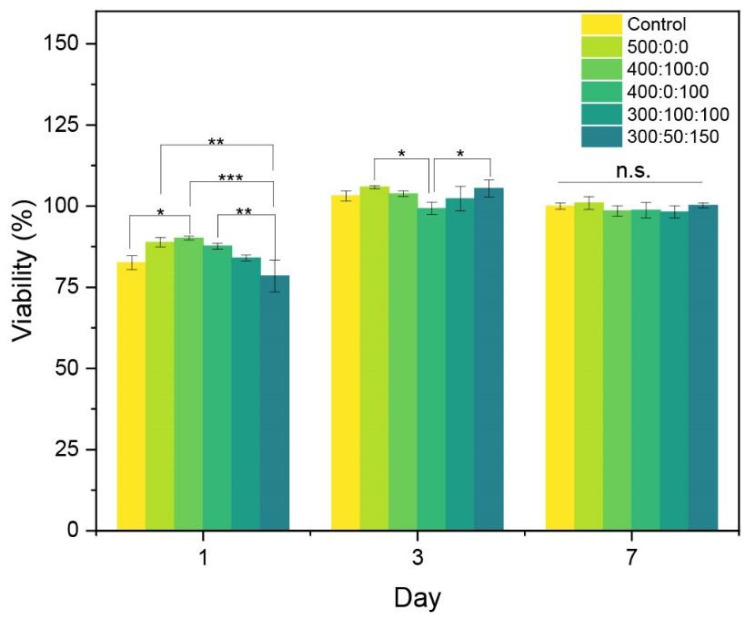
Cell viability results based on the AlamarBlue assay for various resin compositions at 1, 3, and 7 days of culture. Data are expressed as mean ± standard deviation (*n* = 3). * *p* < 0.05; ** *p* < 0.01; *** *p* < 0.001; n.s., nonsignificant differences.

Once the final sample composition was decided and the wrinkle patterns were correctly characterized, the cytocompatibility of the samples was tested. MC3T3-E1 cells were seeded on the material surface, and their viability was monitored for over 7 days. One-way ANOVA statistical analysis was carried out for each group (1, 3, and 7 days) using the null hypothesis that the mean of the distributions was equal. As shown in Figure 8, all samples exhibited high cell viability, with no cytotoxic effects observed. On day 3, a slight decrease in viability across all samples was noted, potentially due to environmental changes in the cell culture. However, this temporary drop did not appear to compromise the materials’ cytocompatibility, as a cell viability increase was recorded by day 7, which implied high proliferation. This recovery suggests that the cells successfully adapted to the substrates, demonstrating stable metabolic activity for at least 7 days. This indicates that our synthesized resins achieve cytocompatibility with over 70% cell viability after 7 days of incubation, compared to the control without material, following the ISO 10993–5 standard for biomedical devices [53].

**Figure 9:**
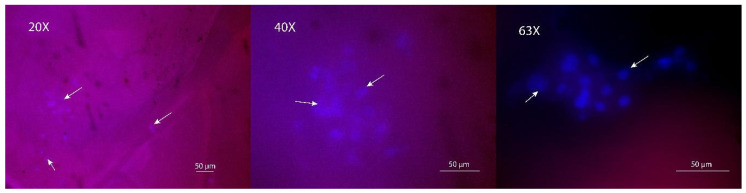
Adhesion of MC3T3-E1 cells on resin surfaces at 24 h of incubation. Bar: 50 µM, magnification: 20×, 40×, and 63×.

In Figure 9, fluorescence microscopy images show the film adhesion and cell spreading on the surface of the 300:50:150 resin. Cells displayed characteristic elongated morphologies at various magnifications, firmly adhering to the substrate (63×). The use of this resin was notable as it exhibited good cell proliferation and adhesion capabilities, further demonstrating the effectiveness of topographical cues in promoting cell adhesion. Wrinkled surfaces have been shown to enhance cell–material interactions by offering physical features that mimic the ECM [54,55]. This microstructural complexity facilitates the cells’ ability to anchor and spread efficiently [55].

## 3. Conclusions

This study developed a methodology for creating 3D porous scaffolds with controlled surface topographies through PET-RAFT polymerization and wrinkling pattern generation over nonplanar surfaces. Scaffolds could be created using high-resolution DLP with photosensitive hydrogel-based resins. The fabrication process included a spin-coating step to create thin layers of material, followed by treatments that resulted in either planar or wrinkled surfaces, enhancing osteogenesis capabilities. Multiple analyses, including surface tension, rheological tests, and mechanical/biological assessments, were conducted to evaluate scaffold properties and ensure compatibility with natural bone.

The characterization of resin printability involves two main tests: rheological testing to ensure appropriate viscosity and measurement of the adhesion forces. Rheologic parameters confirm the formation of a stable liquid bridge through contact angle measurements and Bond number analysis. Parallel, different scaffold designs such as gyroid, straight channels, and solid structures were tested for mechanical performance, revealing that solid models exhibited the highest resistance, as expected, followed by gyroid structures. After the data analysis, it was possible to ascertain that variations in the internal architecture significantly influenced mechanical strength and deformation. AFM and FE-SEM allowed us to characterize the surface of the wrinkled patterns, thus permitting us to select the most appropriate parameter set for spinning conditions. The results indicated that modifying the spin-coating parameters influenced the wrinkle dimensions, enhancing the surface’s functionality.

Finally, the cytocompatibility of the developed scaffolds was evaluated using the MC3T3-E1 cell line. High cell viability was observed, confirming the noncytotoxic nature of the scaffolds and demonstrating that cells proliferated well on the designed surfaces. In conclusion, the selected resin composition (300:50:150) proved suitable for biomedical applications due to its favorable mechanical and improved surface roughness properties, as well as cytocompatibility. This study underscores the significance of surface topography in improving cell–material interactions and guiding future scaffold designs.

## 4. Materials and Methods

### 4.1. Materials

2-(Dimethylamino)ethyl methacrylate (DMAEMA, 97%), acrylamide (AAm, 98%), and poly(ethylene glycol) diacrylate (PEGDA, 99.9%), with an average molecular weight of 575 g mol^−1^, were used as monomers and crosslinking agents, respectively. Additionally, triethanolamine (TEOH, 99%), eosin Y disodium salt (EY, 90%), and Rose Bengal (RB, 80%) served as the reducing agent, photoinitiator, and photoabsorbing agent, respectively. All these chemicals were sourced from Sigma Aldrich (St. Louis, MO, USA). Sigma Aldrich also provided the compound S-S dibenzyl trithiocarbonate (DBTTC, 97% HPLC grade) as the RAFT agent. The same supplier provided glycerol (C_3_H_8_O_3_) and diiodomethane (CH_2_I_2_). On the other hand, ethanol (C_2_H_6_O, 99.9%) and isopropanol (C_3_H_8_O) were procured from Merck (Darmstadt, Germany), and MilliQ water was provided by Symplicity water purification systems (Merck KGaA, Darmstadt, Germany).

For the biological studies, MC3T3-E1 cells (ATCC, Manassas, VA, USA) were cultured in Dulbecco’s modified Eagle medium (DMEM) supplemented with HEPES, penicillin, streptomycin, and amphotericin B (all from Sigma-Aldrich, St. Louis, MO, USA), along with fetal bovine serum (FBS) from Gibco^TM^, ThermoFisher (Waltham, MA, USA). Cell viability was assessed using the AlamarBlue HS^®®^ reagent, also from ThermoFisher.

### 4.2. Equipment

The 3D printing was conducted using a Photon Mono 4K from AnyCubic (Shenzhen, China). The light source was modified to emit a wavelength of 525 nm, using 15 LEDs of 3 W each, totaling 45 W, and a light intensity of 3500–4500 µW/cm^2^. The scaffolds were then cured by exposing them to radiation from a custom-built 525 nm lamp, equipped with 12 LEDs of 3 W each, for a total output of 36 W and approximately 150 mW/cm^2^.

A KW-4 spin-coater (Northridge, Los Angeles, CA, USA) was used to evenly spread the unpolymerized resin across the material surfaces to assess wrinkle formation on planar and nonplanar films. For an initial inspection of the material surface and to confirm wrinkle presence, a ZEISS Axioscope upright optical microscope (Carl Zeiss AG, Oberkochen, Germany), equipped with Axiocam cameras and ZEN core imaging software, version 3.10, was utilized. To further visualize the surface, GeminiSEM 360 field-emission scanning electron microscopy (FE-SEM) (Carl Zeiss AG, Oberkochen, Germany) with a Gemini 1 InLens detector provided high-efficiency signal detection for both secondary (SE) and backscattered (BSE) electrons. To enhance image resolution, the samples were coated with an 8 nm gold layer using a model 108 AUTO argon sputter-coater (Cressington Scientific Instruments Ltd., Watford, UK). Surface topography was then captured with a CoreAFM from Nanosurf Inc. (Woburn, MA, USA) in tapping mode, and images were processed using Gwyddion 2.42 offline freeware.

The rheological assessment of all synthesized resins was conducted using an Anton Paar MCR 72 rheometer (Graz, Austria) with parallel-plate geometry (PP 25, 25 mm diameter) for viscosity measurements under dynamic oscillatory shear tests. A 250 μm gap between plates was maintained at a controlled temperature of 25 °C, utilizing a Peltier-controlled Plate System (CoolPeltier^TM^).

Water contact angle measurements were obtained with a Theta Lite optical tensiometer from Attension-Biolin Scientific (Gothenburg, Sweden), applying 4 μL of liquid (water, glycerol, or diiodomethane) onto the solid sample. The chemical composition of the photopolymerized resins was analyzed using FT-IR on a Nicolet iS-20 spectrometer from ThermoFisher (Waltham, MA, USA), covering a wide spectroscopic range (7800 to 350 cm^−1^).

Mechanical tests were performed using a Zwick–Roell ProLine Z005 universal testing machine (Ulm, Germany) with a 2500 N load cell, operating at a testing speed of 2.5 mm/min (quasistatic regime). Cell viability was measured by absorbance at 570 nm for biological studies using a BioTek Synergy HTX microplate reader from Agilent Technologies Inc. (Santa Clara, CA, USA). Cell cultures were maintained in a ThermoFisher Forma^TM^ Series 3 Water Jacketed CO_2_ Incubator (184 L) from Waltham, MA, USA.

### 4.3. Methodology

#### 4.3.1. Scaffold Design

Different cylindrical scaffolds were prepared with a 10 mm diameter and 10 mm height with three distinct internal structures: a TMPS-generated structure with 40% porosity (60% of solid material relative to the total volume of the CAD design), a scaffold with straight cylindrical channels maintaining 40% porosity, and a solid scaffold (0% porosity) for comparative purposes.

In addition to the 3D scaffolds, flat circular pieces (10 mm diameter and 0.5 mm thickness) were produced as prototypes for printing parameter optimization and for carrying out cytocompatibility tests.

#### 4.3.2. Design of the Cytocompatible Photosensitive Resins

Five different resins were prepared by varying the concentrations of the crosslinking agent and the monomers. Thus, all the resins had the same components but different relative concentrations: DMAEMA and AAm were employed as monomers, PEGDA_575_ as the crosslinking agent, and DBTTC as the RAFT agent. Table 4 outlines the composition of each resin studied. Before use, the crosslinking agent and monomers were purified with a basic alumina column to eliminate hydroquinone, which is an inhibitor during compound storage. Eosin Y served as the photoinitiator, Rose Bengal (RB) was used as a photoabsorber to enhance printing resolution and accuracy, and TEOH acted as a reducing agent for both reaction mixtures. These latter compounds remained unchanged across all formulations and tests.

All the resins were vortexed and sonicated until complete dissolution was achieved [29]. In each case, these resins were mixed with the exact amounts of photoinitiator, photoabsorber, reducing agent, and RAFT agent to then mix them vigorously.

#### 4.3.3. Evaluation of the Resin Printability

Several aspects can be characterized to evaluate the printability of the resins depending on their composition. Still, the resin must fulfill a fundamental requirement: high printability, which is a property of materials that refers to their suitability to be used as an “ink” in 3D printing.

For this purpose, two different experiments were carried out, i.e., to determine the contact angle between the resin and the metallic plate and the calculation of the so-called Bond number (B_0_), which depends, in turn, on the resin’s viscosity, density, and surface tension.

##### Contact Angle and Surface Tension Measurements

Contact angle measurements were performed by adding 4 μL of the liquid resin (unpolymerized) over the printing platform (as solid phase). In parallel, to obtain the surface free energy (SFE) of the hydrogel-based resins, contact angle measurements were performed over the solid samples (completely polymerized) using 4 μL of water, glycerol, and diiodomethane as liquid phases. As those liquids present different polarities and surface tensions, the Owens, Wendt, Rabel, and Kaelble (OWRK) method was applied to obtain the SFE using the following equation [30]:

(2) $$\frac{\gamma_{L}(\cos\theta +1)}{2{\left(\gamma_{L}^{d}\right)}^{1/2}}={\left(\gamma_{S}^{p}\right)}^{1/2}\frac{{\left(\gamma_{L}^{p}\right)}^{1/2}}{{\left(\gamma_{L}^{d}\right)}^{1/2}}+{\left(\gamma_{S}^{d}\right)}^{1/2}$$

where γ corresponds to the surface tension; the subscripts L and S correspond to the liquid and solid phase, respectively; and the p and d superscripts correspond to the polar and dispersive interactions of the surface tension, respectively. To further understand the behavior of the fabricated resins during the printing process, surface tension was measured using the pendant drop method, with the resin serving as the solid phase and air as the light phase.

#### 4.3.4. Optimization of the 3D Printing Parameters

Once the resins were completely homogenized, the solutions were poured into the 3D-printer vat. The printing experimental conditions, including layer exposure time, initial layer thickness, and number of initial layers, were adjusted in each case and are depicted in Table 5; only the number of bottom layers (8 layers) and the off-time between layers (10 s) were maintained unaltered for the fabrication for all the resins employed.

#### 4.3.5. Formation of Wrinkles: Characterization of Nonplanar Surfaces Topography

When the scaffolds (solid, cylindrical pores, and gyroid) were successfully printed, four sets of parameters were tested to generate wrinkles on the surfaces of the scaffolds using the spin-coating technique. The as-printed pieces were removed from the platform and placed in the spin-coater without further treatment (uncured), varying the time and spin velocity (Table 6). The spin-coating process was carried out to generate a homogenous thin film (constant thickness) of the uncured remanent resin (liquid) on the scaffold surface. This process was carried out either using one or two cycles: the first one corresponded to a slow rotation, which slightly distributed the liquid resin, and the second one which was fast enough to remove the excess unpolymerized resin.

Once the thin liquid layer of resin was obtained, green light was used to polymerize the material (30 s). Then, vacuum (2 h) and argon plasma processes (20 s) were used to trigger the formation of spontaneous wrinkled patterns (see Figure 1). A final posturing step was carried out by exposure for 2 h to green light to ensure complete monomer conversion. Finally, for those scaffolds to be employed in the biological tests after printing and wrinkling formation, the pieces were washed using distilled water for 20 h and postcured using a green lamp (36 W, 525 nm) for 2.5 h.

#### 4.3.6. Chemical Characterization of the Photopolymerization Step

The chemical composition of the printed scaffolds was characterized via FT-IR, using the liquid (unpolymerized resin) and the cured (completely polymerized) materials. The chemical structure was studied with an FT-IR Nicolet iS 5 in the attenuated total reflection (ATR) mode, with a wavelength range from 500 to 4000 cm^−1^.

#### 4.3.7. Mechanical Compression Strain–Stress Tests

Mechanical compression tests were performed on the different scaffolds to obtain stress–strain curves and to evaluate key mechanical properties of the 3D-printed parts, such as stiffness (Young’s modulus) and maximum fracture load. The cylindrical specimens were measured prior to testing, with initial dimensions defined by length (L_0_) and radius (R_0_). The applied load (F) and the displacement (Δ) were recorded during testing. The axial stretch (λ) could then be determined using the following equation:

(3) $$\lambda =\frac{L_{0}+\Delta }{L_{0}}$$

Then, the engineering stress could be calculated as follows:

(4) $$\sigma =\frac{F}{\pi R_{0}^{2}}$$

The initial test conditions were a preload 5 N and speed ~2 mm/min (strain rate 0.1%) until fracture. In our case, the tested cylindrical scaffolds’ initial dimensions were approximately L_0_ = 10 mm and R_0_ = 5 mm. It is important to mention that these values corresponded to the ideal dimensions that the piece should have had according to the CAD model, but they were not equal to the real dimensions obtained after printing. This is why it was essential to measure the real dimensions of each one of the pieces before testing them mechanically. The real values differed by approximately ~1% from the ideal dimensions.

#### 4.3.8. Evaluation of In Vitro Cytocompatibility

MC3T3-E1 cells (ATCC, UK) were cultured in DMEM (which contained 10% FBS, 10 mM HEPES, 100 U/mL penicillin, 100 µg/mL streptomycin, and 2.5 µg/mL amphotericin B). A total of 5 × 10^4^; cells were seeded onto a polymeric 3D-printed disk placed in a 48-well plate. The culture medium was refreshed every three days, and cell viability was assessed at 1, 3, and 7 days using the AlamarBlue HS^®®^ assay to measure cell metabolic activity. After 2 h of incubation with AlamarBlue at 37 °C in a 5% CO_2_ humidified atmosphere, the medium was collected, and absorbance was measured at 570 nm with a microplate reader.

For imaging, MC3T3-E1 cells were seeded onto coverslip-mounted samples (12 mm diameter), rinsed with phosphate-buffered saline (PBS; 10 mM Tris, pH 7.4, 100 mM NaCl, 5 mM KCl), and fixed in 4% paraformaldehyde for 15 min at RT. Cells were washed thrice with PBS, permeabilized with 0.5% Triton X-100 for 10 min, and stained with Phalloidin (Cytoskeleton, Inc., Denver, CO, USA) for 30 min. The slides were air-dried in the dark and mounted with Duolink in situ mounting medium with DAPI (Sigma, DUO82040) for 15 min. Finally, the cells were visualized with a Zeiss–Colibri epifluorescence microscope using 20×, 40×, and 63× objectives, and images were analyzed with Fiji software (ImageJ, version 1.54i, 2024).

## Figures and Tables

**Table 1:** The parameters of the physicochemical properties of the photosensitive resins selected for this work.

Mole Ratio	Dynamic Viscosity (mPa × s)	Surface Tension (mN/m)	Density (Kg/m^3^)	Bond Number
500:0:0	20.16 ± 2.5	53.55 ± 5.6	1100	3.2 × 10^^−5^^
400:100:0	23.44 ± 5.1	50.08 ± 4.9	1060	3.3 × 10^−5^
400:0:100	25.42 ± 6.2	54.81 ± 0.19	1106	3.1 × 10^−5^
300:100:100	24.35 ± 6.1	50.51 ± 5.3	1070	3.1 × 10^−5^
300:50:150	24.21 ± 4.1	53.14 ± 0.11	1050	3.0 × 10^−5^

**Table 2:** Mechanical parameters (Young modulus, stress, and strain at fracture) were obtained from compression tests for solid, straight channels, and gyroid structures for sample 300:50:150.

	Young Modulus (MPa)	Stress @ Fracture (MPa)	Strain @ Fracture (%)
Solid	9.08 ± 0.56	24.70 ± 3.09	3.2 ± 0.83
Gyroid	0.51 ± 0.05	17.08 ± 2.97	66.8 ± 2.03
Straight Channels	0.36 ± 0.07	5.53 ± 2.45	27.7 ± 1.35

**Table 3:** Contact angle, surface tension, and SFE for resin 300:50:150.

Contact Angle (°)				Surface Tension (Dyne/cm)		SFE (Dyne/cm)
	Water	Glycerol	Diiodomethane	γ_SD_	γ_SP_	
Smooth	47.69 ± 1.23	55.40 ± 0.96	24.49 ± 0.45	15.24	39.51	54.57
Wrinkled	25.50 ± 0.78	45.35 ± 1.12	13.38 ± 1.02	23.90	40.99	64.89

**Table 4:** Different resins tested with their respective composition mole ratio *.

Mole Ratio	PEGDA_575_ (g)	DMAEMA (g)	AAm (g)
500:0:0	11.00	0.00	0.00
400:100:0	8.88	0.60	0.00
400:0:100	8.88	0.00	0.27
300:100:100	6.66	0.67	0.27
300:50:150	6.66	0.30	0.41

* All the resins possessed the following components: DBTTC: 11 mg, TEOH: 110 mg, EY: 0.26 mg, RB: 0.26 mg.

**Table 5:** Optimized 3D-printing parameters employed for the fabrication of the scaffolds.

Mole Ratio	Bottom Layer Exposure (s)	Normal Layer Exposure (s)	Layer Thickness (µm)
500:0:0	200	200	100
400:100:0	200	150	100
400:0:100	150	120	100
300:100:100	150	120	100
300:50:150	150	120	25

**Table 6:** Different first and second spin-coating cycles (velocities and times) were used for thin-film coating generation on the surface of the scaffolds.

Coating Parameters Code	1st Cycle (s/RPM)	2nd Cycle (s/RPM)
**1**	6/1000	-
**2a**	10/1250	-
**2b**	10/1250	10/1500
**3a**	10/1500	-
**3b**	10/1500	10/2000
**4a**	10/1500	-
**4b**	10/1500	10/3000

## Data Availability

Data are contained within the article.
